# Mental health knowledge, attitudes, and self-efficacy among primary care physicians working in the Greater Tunis area of Tunisia

**DOI:** 10.1186/s13033-018-0243-x

**Published:** 2018-10-26

**Authors:** Jessica Spagnolo, François Champagne, Nicole Leduc, Michèle Rivard, Myra Piat, Marc Laporta, Wahid Melki, Fatma Charfi

**Affiliations:** 1 School of Public Health, IRSPUM, University of Montreal, Montreal, QC H3N1X9 Canada; 2 School of Public Health, University of Montreal, Montreal, QC Canada; 3 Douglas Mental Health University Institute, McGill University, Montreal, QC Canada; 4 Montreal WHO-PAHO Collaborating Center for Research and Training in Mental Health, McGill University, Montreal, QC Canada; 5 Razi Hospital, University of Tunis El-Manar, Tunis, Tunisia; 6 Mongi-Slim Hospital, University of Tunis El-Manar, Tunis, Tunisia

**Keywords:** Mental health, Physicians, Primary care, Knowledge, Attitudes, Self-efficacy, Tunisia

## Abstract

**Background:**

Non-specialists’ involvement in mental health care is encouraged in the field of global mental health to address the treatment gap caused by mental illness, especially in low- and middle-income countries. While primary care physicians (PCPs) are involved in mental health care in Tunisia, a lower-middle-income country in North Africa, it is unclear to what extent they are prepared and willing to address mental health problems, substance use disorders, and suicide/self-harm. In this context, we aim (1) to report on mental health knowledge, attitudes, and self-efficacy among a sample of PCPs working in the Greater Tunis area, prior to the implementation of a mental health training program developed by the *World Health Organization*; and (2) to identify what characteristics are associated with these competencies.

**Methods:**

In total, 112 PCPs completed questionnaires related to their socio-demographic and practice characteristics, as well as their mental health knowledge, attitudes, and self-efficacy. Descriptive analyses and regression models were performed.

**Findings:**

PCPs had more knowledge about depression, symptoms related to psychosis, and best practices after a suicide attempt; had favourable attitudes about distinctions between physical and mental health, learning about mental health, and the acceptance of colleagues with mental health issues; and believed most in their capabilities related to depression and anxiety. However, most PCPs had less knowledge about substance use disorders and myths about suicide attempts; had unfavorable attitudes about the dangerousness of people with mental health problems, personal disclosure of mental illness, non-specialists’ role in assessing mental health problems, and personal recovery; and believed the least in their capabilities related to substance use disorders, suicide/self-harm, and psychosis. Participation in previous mental health training, weekly hours (and weekly hours dedicated to mental health), weekly provision of psychoeducation, and certain work locations were associated with better mental health competencies, whereas mental health knowledge was negatively associated with weekly referrals to specialized services.

**Conclusions:**

Findings suggest that PCPs in our sample engage in mental health care, but with some gaps in competencies. Mental health training and increased interactions/involvement with people consulting for mental health issues may help further develop non-specialists’ mental health competencies, and integrate mental health into primary care settings.

**Electronic supplementary material:**

The online version of this article (10.1186/s13033-018-0243-x) contains supplementary material, which is available to authorized users.

## Background

Non-specialists’ involvement in mental health care is a vision upheld in the field of global mental health to address the alarming treatment gap caused by mental, neurological, and substance use (MNS) disorders, which are especially elevated in low- and middle-income countries (LMICs) [1–4]. A non-specialist is defined as “any type of health worker (like a doctor, nurse, or lay health worker) who is not a specialist in mental health or neurology but who may have had some training in these fields” [5]. International efforts currently encourage and reinforce the use of non-specialists in mental health care because it is common for them to already be involved in mental health detection, treatment, and management, especially in LMICs where mental health providers are limited and/or unevenly distributed within countries [5–8]. Also, the involvement of non-specialists in mental health care has been shown to benefit people’s health outcomes, especially for general and perinatal depression, anxiety, post-traumatic stress disorder, and alcohol-use disorders [5, 7, 9].

While the use of non-specialists in mental health care in resource-limited settings shows promise [5, 7, 9], studies highlight important gaps in their mental health literacy (i.e., knowledge, attitudes, and perceived self-efficacy [10]) that cannot be overlooked. First, non-specialists reported lacking specific knowledge about mental illness and suicide [11–15]. While non-specialists do see people presenting with mental health problems in consultation, the majority are not able to list or recognize symptoms attributable to mental illness [16–19]. Interestingly, this is also a reality observed with depression and anxiety [14, 20], despite these being the most frequently reported and seen mental health problems in non-specialized settings [21, 22]. In addition, non-specialists have difficulties identifying medications used in mental health care, such as antidepressants and antipsychotics [12, 20, 23–25].

Second, studies show that the healthcare system, even if non-institutional, is an environment where people living with mental health problems experience stigma [26]. These negative experiences within the healthcare system are attributable in part to healthcare professionals’ stigma against mental illness. For example, studies show how common it is for healthcare workers to believe that people with mental illness are “violent” and “dangerous” [12, 27–30]. Studies also show that stigmatizing views against mental illness encourage healthcare professionals to associate mental illness with personal, moral faults or weakness [13, 14, 31] and describe people consulting for mental health conditions with derogatory terms, such as “crazy” and “mad” [32]. Thus, it is not surprising that due to negative beliefs about people living with mental illness, healthcare professionals are less likely to personally engage with this type of clientele and show empathy [33, 34]. In addition, studies mention that stigma against mental illness even deters medical students from considering “psychiatry” as a preferred specialty [35, 36].

Finally, self-efficacy, a concept first introduced by Bandura [37, 38], is defined as one’s beliefs in his/her capability to succeed in a specific situation or task. Bandura [37] suggests that individuals with higher levels of self-efficacy will invest themselves more in a specific task and are generally more successful than those with lower levels of self-efficacy. In our case, this concept translates into non-specialists’ belief in their capability to successfully detect, treat, and manage mental health issues at the level of primary care [39]. Studies show that non-specialists question their involvement in the field of mental health because they are not confident in their general mental health skills [12, 40, 41]. Lower levels of confidence in mental health skills is reportedly one of the main factors influencing non-specialists’ decisions to refer patients to specialized mental health services [42, 43]. Hence, studies assessing the feasibility and acceptability of using non-specialists in mental health care commonly highlight the need for ongoing mental health training in order to “boost” confidence [44, 45].

Gaps in knowledge, attitudes, and self-efficacy have important clinical implications. Specifically, they may discourage patients from seeking mental health care [46, 47] and limit access to quality interventions [3, 48–50]. Interestingly, if uncovered, these gaps may be used to tailor the content of training programs in order to make them more clinically useful, which is also a way to encourage the further integration of mental health into primary and community-based settings [3, 44, 51, 52].

Tunisia, a lower-middle-income country located in North Africa [53], is among the many countries concerned with the provision of effective mental health care to target the growing mental health treatment gap [23]. This gap is on the rise given widespread untreated mental health symptoms, especially since the 2010–2011 Revolution [23, 54–57]. Lack of treatment is caused, in part, by human resource challenges [23, 39, 58]. First, it is worth highlighting deficits in the availability of trained mental health providers. Psychiatrists are unevenly distributed across the country, creating disparities in care [23, 58]. More specifically, they are mainly located in and around the capital, or along the coastline, despite suicide rates being reported as highest in the interior of the country [23]. In addition, mental health nurses and psychosocial care providers are estimated at 3.7 per 100,000 and 2.9 per 100,000 people respectively, numbers insufficient to meet current need in Tunisia [6]. To address this shortage, the number of needed mental health nurses and psychosocial care providers is projected at 13.7 per 100,000 and 9.8 per 100,000 people respectively [6].

Second, 30–40% of consultations done by PCPs are related to mental health care, making them the most relied upon non-specialist for this type of care in Tunisia [39]. The reason PCPs receive many mental health consultations is a consequence of attempts made in the 1990s to integrate mental health care within primary health centers, which provide outpatient care, including preventative and curative health services, as well as health education [58]. Even though this integration was done non-systematically and with limited follow-up, it was a way to ensure access to mental health care for the majority [58]. However, these attempts to integrate mental health care within primary health centers were (and still are) challenged, in part, by issues with continuing mental health training in Tunisia. While mental health training programs have been offered to primary care physicians in Tunisia, these were not offered as part of a systematic national program. Thus, previous mental health training programs were offered under the leadership of individual governorate directors, which limited national efforts to further integrate mental health into existing primary and community-based services [23, 39, 58]. Therefore, while PCPs are (and are encouraged to be) involved in the care of people living with MNS disorders in Tunisia [23, 39], little is known about their preparedness and willingness to address mental health problems, substance use disorders, and suicide/self-harm in primary care in Tunisia. We identified a few studies that did help shed light on this topic. For example, a study conducted on attitudes towards schizophrenia among randomly selected PCPs in the Greater Tunis area suggests that most underestimated the prevalence of schizophrenia, and 48.5% were incapable of naming medications for use in first episode psychosis [24]. These findings corroborate with those of a study conducted in central Tunisia, which suggest that 53% of PCPs did not master the prescription of antipsychotic medications [23, 25].

This paper is part of a pilot trial that seeks to contextualize, implement, and evaluate a mental health training program based on the *Mental Health Gap Action Programme* (*mhGAP*) *Intervention Guide* (*IG*) [2] developed by the *World Health Organization* (*WHO*). More specifically, the pilot trial aims to implement and evaluate the tailored training program offered to PCPs working in the Greater Tunis area to further the development of proximity mental health services [23, 39, 59]. The training includes the following modules, chosen by members of the Tunisian Ministry of Health to meet the most pressing mental health needs in the country: general principles of care, depression, psychosis, suicide/self-harm, and alcohol and drug use disorders.

The two aims of this paper are: (1) to report on mental health knowledge, attitudes, and self-efficacy among a sample of PCPs working in the Greater Tunis area of Tunisia prior to their involvement in the training program; and (2) to identify what characteristics are associated with these competencies. Uncovering such information is useful for informing mental health training material that targets non-specialists working in the area and for formulating aspects of health policy.

## Methods

### Sample and setting

The sample consisted of PCPs working in primary care in the Greater Tunis area, a setting divided into four governorates: Tunis, Manouba, Ben Arous, and Ariana. Manouba, Ben Arous, and Ariana are also referred to as the “suburbs” of Tunis. The Greater Tunis area was chosen for the pilot trial because its setting diversity is representative of other areas in Tunisia. For example, Tunis is considered urban, Ben Arous, rural and semi-urban, and Ariana, rural and urban. Manouba, where the only operating mental health hospital is located, is considered rural and semi-urban.

Recruitment was facilitated by physicians working in the Greater Tunis area who were involved in organizing continuing medical education in this area. They compiled a list including 345 PCPs, all of whom were part of the primary care physicians’ professional order in Tunisia, worked in the public sector, and previously attended continuing medical education training in the Greater Tunis area. Continuing medical education is highly recommended and encouraged in Tunisia, specifically for the advancement of PCPs’ careers. Therefore, we believe that this list regroups all PCPs working in the public sector in the Greater Tunis area. Of these, 315 met the following study eligibility criteria: (1) working at the level of primary care in the Greater Tunis area; and (2) having five or more years of clinical experience.

Physicians, a psychiatrist involved in the recruitment of participants given her ties to community mental health, and JS proceeded to contact the 315 PCPs. One hundred thirty-two PCPs (41.90%) accepted to participate in the trial. The others (n = 183) were not included in the trial for the following reasons: unavailability or not being reached for recruitment. To obtain consent, JS contacted the 132 PCPs who accepted to participate in the trial at the beginning of January 2016. Once consent was obtained, they were asked to complete a baseline questionnaire by the end of January 2016, a date prior to the implementation of the training. From the time consent was obtained until this deadline, JS sent reminder emails and made calls to PCPs who did not complete the questionnaire. These reminders were done once a week, for 2 weeks. One hundred and twelve (n = 112) PCPs met the deadline to submit the questionnaire and were thus included in the larger pilot trial.

### Data collection

Before the training, PCPs were invited to complete self-administered questionnaires on socio-demographic and practice characteristics, mental health knowledge, attitudes, and perceived self-efficacy. All questionnaires were administered in French but were verified prior to distribution by two French-speaking people who had knowledge of general and medical terms used in Tunisia. The questionnaires were then pilot tested on a sample of ten Tunisian healthcare professionals (three trainer-psychiatrists and seven PCPs in charge of continuing medical education in the Greater Tunis area) to identify unclear or confusing items. Questionnaires took 20 min on average to complete.

#### Participant socio-demographic and practice characteristics

We collected demographic information for each PCP, including data on age, gender, country of origin, mother tongue, and medical school location. Practice characteristics included work location (i.e., governorate), number of years working as a PCP, number of work hours per week, and mental health training in the past 12 months (i.e., January 2015–January 2016). We also asked PCPs to report on their total number of patients seen per week, including those presenting with mental health problems; total number of consultations for mental health problems made with and without appointment each week; total number of hours per week allocated to mental health practice; consultations with patients for specific mental health conditions per week; types of treatment provided to patients presenting with mental health problems per week; and frequency of follow-up provided to patients presenting with mental health problems.

#### Knowledge

The knowledge questionnaire was developed by the WHO to accompany the training package [2]. Given its unavailability to the research team in French prior to data collection, the English version was translated into French, and was verified by two members of the WHO office in Tunisia. The questionnaire we used contained sixteen questions, nine being multiple choice and seven True/False. The questions related to material in the training program, and included questions on general principles of care, depression, psychosis, suicide/self-harm, and drug/alcohol use disorders. Questions were grouped into sub-themes to capture information about knowledge on specific training modules, pharmacological and non-pharmacological treatments, manifestation of various mental illnesses, and the management of these mental illnesses. Correct answers were scored as 1 and incorrect answers as 0. A participant’s score is therefore the sum of correct answers for individual items. The authors converted the overall and sub-theme scores to a score ranging from 0 to 10. A higher score indicates more knowledge on topics related to mental health and illness, while a lower score indicates more gaps in knowledge.

Test–retest reliability considers the temporal stability of a measure at two different time points [60]. The Intraclass Correlation Coefficient (ICC) [60, 61] was assessed among 47 individuals. They were randomly assigned to the control group of our trial and thus completed two pre-test measures, 6 weeks apart. According to suggested cut-off [61], a good degree of reliability was found between the two pre-test measures: the average measure ICC was .708, with a 95% confidence interval (CI) [.478 to .837].

#### Attitudes

To measure attitudes towards mental illness and the field of mental health, the *Mental Illness: Clinicians’ Attitudes (MICA) Scale* (version 4.0) was used [62, 63]. The scale has sixteen items, with answers ranging on a six-point Likert scale. For statements 3, 9, 10, 11, 12, and 16, items were scored as follows: ‘strongly agree’ = 1; ‘agree’ = 2; ‘somewhat agree’ = 3; ‘somewhat disagree’ = 4; ‘disagree’ = 5; and ‘strongly disagree’ = 6. All other items were reverse-scored. Scores on individual items were summed to obtain the overall score for each participant within a range of 16–96 points. A higher global score indicates a more negative perception of mental illness and the field of mental health.

We chose the *MICA*-*4* because it was found to be reliable in a sample of nursing students [62]. Analysis revealed that the overall scale had good internal consistency (Cronbach’s alpha = .720) and item-total correlations (at least .2), representing an acceptable fit. To complement these psychometric properties, the scale’s authors suggest considering the applicability of the *MICA*-*4* across other samples by verifying the Cronbach’s alpha and assessing the scale’s test–retest reliability [62]. We were able to assess both of these psychometric properties in our sample.

The Cronbach’s alpha for all sixteen items of the *MICA-4*, when applied to our sample, was .521, which is considered poor [64, 65]. To increase the scale’s internal consistency, we sequentially removed items with an item-total correlation of less than .2 [66] and reassessed the scale’s Cronbach’s alpha. The complete results of this procedure are illustrated in Additional file 1 accompanying this paper. We assessed the item-total correlations of the original sixteen-item scale. At first, question 6 was removed because it was uncorrelated to other items (i.e., showing a negative result), unsurprising given that participants expressed difficulties with this question during the diffusion of preliminary results. However, the healthcare professionals on whom the questionnaire was pilot tested did not mention any issues with this question. The removal of questions 6 increased the scale’s Cronbach’s alpha to .552 (Test 1) and allowed us to consider the removal of question 11, as it yielded the lowest value for item-total correlations and would increase the scale’s Cronbach’s alpha to .563 (Test 2). With question 11 removed, question 3 yielded the lowest value for item-total correlations. Its removal increased Cronbach’s alpha to .573 (Test 3). With question 3 removed, question 8 yielded the lowest value for item-total correlations. Its removal increased Cronbach’s alpha to .598 (Test 4). The removal of question 8 caused questions 9 and 12 to have the lowest values for item-total correlations. We decided to keep question 12 (i.e., “the public does not need to be protected from people with a severe mental illness”) because its content focuses on one of the most commonly measured components of public stigma: belief in the dangerousness of people with a mental disorder [67]. In addition, Table 3 shows that question 12 yielded the least favorable answers among our sample; thus, it has the greatest potential for change post-training. We therefore removed question 9. Not only did it yield one of the lowest values for item-total correlations, but it also increased the scale’s Cronbach’s alpha to .608 (Test 5).

In sum, we report on eleven questions of the *MICA*-*4* (i.e., 1, 2, 4, 5, 7, 10, 12, 13, 14, 15, and 16), which yielded a Cronbach’s alpha of .608 (Test 5). We deemed this value appropriate; even though Cronbach’s alpha is a function of scale length [65], it increased in our case by removing items from the original scale. To compute the overall score for the eleven questions we used for the purposes of this paper, scores on individual items were summed for each participant, yielding a value between 11 and 66. A higher global score indicates a more negative perception of mental illness and the field of mental health.

The ICC [60, 61] for the eleven questions of the *MICA*-*4* was assessed among 47 individuals randomly assigned to the control group of our trial. They completed two pre-test measures, 6 weeks apart. According to the suggested cut-off [61], a good degree of reliability was found between the two measures: the average measure of the ICC was .704 with a 95% CI [.468 to .835].

#### Self-efficacy

The self-efficacy questionnaire was developed in French for the purposes of the pilot trial because Bandura (2006) [38] suggests that the best way to measure self-efficacy is by constructing specific scales per tasks to be explored. Hence, we developed a questionnaire through which we aimed to understand PCPs’ judgement of their capabilities related to detecting depression, psychosis, suicide/self-harm, and alcohol/drug use disorders, using detection techniques (scale 1, range 0–40), and treating and managing patients who present with these disorders (scale 2, range 0–100).

Scale 1 has ten items and scale 2, twenty-five items (for a total of thirty-five questions on the overall questionnaire), with answers ranging on a five-point Likert scale. Each statement was scored as follows: ‘strongly agree’ = 0; ‘somewhat agree’ = 1; ‘neutral’ = 2; ‘somewhat disagree’ = 3; and ‘strongly disagree’ = 4. For scale 1, items were regrouped into two themes: capabilities to detect mental health problems (six questions) and capabilities to use techniques related to detecting mental health problems (four questions). For scale 2, items were regrouped into the following themes: capabilities to provide treatment by pharmacology (five questions), treatment by support (i.e., active listening or psychosocial support) (seven questions), and treatment by psychoeducation (five questions), as well as confidence in capabilities to manage mental health problems in primary care, mainly by developing clinical plans (eight questions). Participants’ overall and sub-theme scores were the sum of correct answers for individual items. Overall and sub-theme scores were converted to a score ranging from 0 to 10. A higher score indicates more confidence in capabilities to detect, treat, and manage mental health problems in primary care, while a lower score indicates more gaps in self-efficacy.

Regarding sub-themes for scale 1, the value of Cronbach’s alpha was .831 for the theme of detecting mental health problems and .791 for the theme of using techniques related to detecting mental health problems. Regarding scale 2, the value of Cronbach’s alpha was .770 for the theme of pharmacological treatment, .868 for the theme of treatment by support (i.e., active listening or psychosocial support), .870 for the theme of treatment by psychoeducation, and .882 for the theme of management of mental health conditions. The Cronbach’s alphas for these themes were satisfactory [64, 65].

The ICC [60, 61] for the self-efficacy scale was assessed among 47 individuals randomly assigned to the control group of our trial. They completed two pre-test measures, 6 weeks apart. According to the suggested cut-off [61], a good degree of reliability was found between the two measures: the average measure ICC was .781 with a 95% CI [.606 to .878].

### Data analyses

All analyses were conducted using SPSS version 25.0 [68]. Incorrect answers on the knowledge questionnaire were reported per question and sub-theme. For reporting answers of the *MICA*-*4*, suggested answers were reported as a single category of “favorable answers.” More specifically, for reverse-scored items, suggested answers tend toward the negative (i.e., ‘strongly disagree’ and ‘disagree’). These negative categories were thus collapsed into the single category of “favorable answers.” Contrarily, for items not reversed, suggested answers tend toward the positive (i.e., ‘strongly agree’ and ‘agree’). These positive categories were thus collapsed into the single category of “favorable answers.” For reporting answers of the self-efficacy questionnaire, categories of “agree” (i.e., ‘strongly agree’ and ‘somewhat agree’) were collapsed and reported. If participants were missing more than 20% of the data on the mental health knowledge, attitudes, or self-efficacy questionnaires, their individual scores were excluded from the overall respective scale score. This resulted in excluding two participants’ scores from the self-efficacy questionnaire’s baseline overall score.

For descriptive analyses, group frequencies and percentages were reported for categorical variables. Means (M), standard deviations (SD), as well as quartiles 1 (Q1), 2 (Q2—the median), and 3 (Q3) were reported for continuous variables.

To assess the association between socio-demographic/practice characteristics and mental health knowledge, attitudes, and self-efficacy, simple linear regression models were performed. Several steps were involved in undertaking such analyses. First, categorical variables were coded using dummy coding to include them in regression models [69]. Second, to respect the assumption of normality, we applied square root, logarithmic, or reciprocal (inverse) transformations [69] to highly skewed practice characteristics not normally distributed prior to conducting these models. Competency variables (i.e., knowledge, attitudes, and self-efficacy) were normally distributed. Third, once non-normally distributed data was transformed, correlation analyses were used to examine the correlation structure between socio-demographic/practice variables. Strong associations between variables may suggest that they provide the same type of information. Two variables were omitted from the regression models, given their high association: the variable “average number of years working as a PCP,” which had a high association with PCPs’ age (*r* = .780), and the variable “average number of consultations for mental health without appointment,” which had a high association with “average number of consultations for mental health per week” (*r* = .869). Last, simple linear regression models were run to assess the association between each socio-demographic/practice characteristic and levels of mental health knowledge, attitudes, and self-efficacy. Unstandardized beta coefficients (*B*), *p*-values, and coefficients of determination (*r*^*2*^) were reported for statistically significant associations. Two-tailed *p*-values of less than .05 were considered statistically significant.

## Results

Data was collected by self-administered questionnaires in January 2016, prior to implementation of the training.

### Participant socio-demographic and practice characteristics

As shown in Table 1, most PCPs included in the sample were born in Tunisia, spoke Arabic as a mother tongue, were women, attended medical school in Tunisia, and worked full-time. Mean average age of participants was 49 years of age, and they had worked on average 17.8 years as a PCP. Few PCPs reported having any mental health training in the last 12 months (i.e., January 2015–January 2016).

**Table 1:** Primary care physicians’ socio-demographic and practice characteristics (n = 112)

Characteristics	Continuous variables	Categorical variables
Socio-demographic characteristics	M (SD) (Q1, Q2, Q3)	n (%)
Age (in years)	49.0 (5.5) (46.0, 49.0, 53.0)	–
Women	–	90 (80.4)
Born in Tunisia^b^	–	109 (97.3)
Mother tongue, Arabic^b^	–	111 (99.1)
Medical school in Tunisia^b^	–	104 (92.9)
Practice characteristics	M (SD) (Q1, Q2, Q3)	n (%)
Governorate—n (%)		
Tunis	–	43 (38.4)
Ariana	–	28 (25.0)
Manouba	–	21 (18.8)
Ben Arous	–	20 (17.9)
Average number of years working as a PCP^c^	17.8 (6.0) (15.0, 18.0, 21.8)	–
Hours work/week	34.1 (5.1) (30.0, 36.0, 36.0)	–
Mental health training in the last 12 months (yes)	–	14 (12.5)
Average number of patient consultations/week	145.3 (57.8) (103.8, 138.5, 180.0)	–
Average number of consultations for mental health/week	17.7 (19.8) (5.0, 12.0, 21.1)	–
Average number of consultations for mental health/week:		–
By appointment^a^	3.3 (8.1) (.0, .5, 3.0)	
Without appointment^a,d^	14.8 (18.7) (3.5, 9.8, 18.0)	
Average number of hours dedicated to mental health care/week^a^	4.5 (3.8) (2.1, 3.6, 6.0)	–
% of mental health consultations per week according to diagnosis: types of mental health consultation per week:		
Anxiety	49.5 (25.5) (30.0, 50.0, 70.0)	–
Depression	33.0 (22.3) (20.0, 30.0, 45.0)	–
Alcohol use disorders	8.8 (14.5) (.0, 3.0, 10.0)	–
Drug use disorders	6.6 (13.5) (.0, 2.0, 10.0)	–
Psychosis (including schizophrenia)	5.1 (7.9) (.0, 2.0, 9.0)	–
Suicide/self-harm	3.7 (7.9) (.0, 1.0, 5.0)	–
% of mental health clientele:		
Referred to specialized care^a^	55.6 (30.8) (30.0, 50.0, 80.0)	–
Receiving support (ex.: active listening)	51.8 (36.9) (20.0, 50.0, 90.0)	–
Receiving psychoeducation	40.7 (38.4) (.0, 35.0, 80.0)	–
Receiving pharmacology	39.6 (36.3) (5.0, 30.0, 80.0)	–
Receiving psychotherapy	18.7 (29.0) (.0, 1.0, 23.8)	–
Average number of follow-up visits/patients with mental health issues^a^	7.1 (8.8) (4.0, 4.0, 6.0)	–

^a^Missing values were greater than 5%, but less than 10%

^b^The variable is not considered in further analyses given the small number of participants in some groups

^c^This variable is not considered in further analyses given the high correlation with the variable ‘age’

^d^This variable is not considered in further analyses given the high correlation with the variable ‘average number of consultations for mental health per week’

PCPs estimated that they saw on average 145 patients per week, approximately 17 of which consulted for mental health issues. The PCPs in our sample reported seeing very few patients consulting for mental health issues by appointment. Per week, they primarily provided consultation for anxiety and depression and mostly referred patients to specialized mental health services or provided support, such as active listening. PCPs followed up with their patients consulting for mental health issues on average roughly seven times a year.

### Knowledge of mental illness

Prior to the implementation of the mental health training in the Greater Tunis area, PCPs obtained an average overall score of 6.5/10 (SD = 1.4; Q1 = 5.6, Q2 = 6.3, Q3 = 7.5) on the knowledge questionnaire. Average scores were highest for sub-themes on general knowledge of depression (7.9/10, SD = 1.8; Q1 = 6.0, Q2 = 8.0, Q3 = 10.0) and psychosis (7.5/10, SD = 2.7; Q1 = 5.0, Q2 = 10.0, Q3 = 10.0), in comparison with sub-themes on knowledge of pharmacological treatment (6.7/10, SD = 3.0; Q1 = 3.3, Q2 = 6.7, Q3 = 10.0), management of mental illness (6.6/10, SD = 2.3; Q1 = 4.0, Q2 = 6.7, Q3 = 8.3), manifestation of mental illness (6.5/10, SD = 1.8; Q1 = 5.0, Q2 = 6.7, Q3 = 8.3), self-harm/suicide (6.1/10, SD = 2.6; Q1 = 5.0, Q2 = 5.0, Q3 = 10.0), non-pharmacological treatment (5.5/10, SD = 2.1; Q1 = 3.3, Q2 = 6.7, Q3 = 6.7), and substance use disorders (3.7/10, SD = 2.8; Q1 = 3.3, Q2 = 3.3, Q3 = 6.7). These results suggest gaps in knowledge about mental health.

Gaps are also made apparent when looking at incorrectly answered questions on the knowledge questionnaire. As shown in Table 2, most physicians responded incorrectly to questions pertaining to the following concepts: identifying symptoms related to alcohol use disorders; acknowledging myths about suicide attempts; effectiveness of brief advice to people with alcohol use disorders; and managing people with drug use disorders.

**Table 2:** Incorrect responses to knowledge statements about mental health and illness (n = 112)

Knowledge of specific mental health conditions and illness manifestation	Incorrect responses: n (%)
Depression	
Administering antidepressants	52 (46.4)
Depression is always treated with antidepressants	39 (34.8)
Severe chronic depression in a mother and repercussions on children	20 (17.9)
Symptoms of depression	5 (4.5)
Advice for people living with depression	4 (3.6)
Substance use disorders	
Symptoms of alcohol use	84 (75.0)
Brief advice to people with alcohol problems is effective	67 (59.8)
Drug use	62 (55.4)
Psychosis	
Interventions for people with acute psychosis	46 (41.1)
Symptoms of psychosis	11 (9.8)
Suicide/self-harm	
Myths about suicide	81 (72.3)
Best practice after a suicide attempt	6 (5.4)
Manifestation of mental illness	
Symptoms of alcohol use	84 (75.0)
Myths about suicide	81 (72.3)
Prevalence of mental illness in youth	31 (27.7)
Severe chronic depression in a mother and repercussions on children	20 (17.9)
Symptoms of psychosis	11 (9.8)
Symptoms of depression	5 (4.5)

Knowledge on provision of care	Incorrect responses: n (%)
Non-pharmacological	
Myths about suicide	81 (72.3)
Brief advice to people with alcohol problems is effective	67 (59.8)
Advice for people living with depression	4 (3.6)
Pharmacological	
Administering antidepressants	52 (46.4)
Depression is always treated with antidepressants	39 (34.8)
Pharmacological treatment for people with mental illness	21 (18.8)
Management of mental illness in primary care	
Drug use	62 (55.4)
Involvement of people with mental illness in their own care	46 (41.1)
Interventions for people with acute psychosis	46 (41.1)
Best place to care for people with mental illness	45 (40.2)
Pharmacological treatment for people with mental illness	21 (18.8)
Best practice after a suicide attempt	6 (5.4)

A total of 112 PCPs completed the questionnaire and there is no missing data. Some items are included in more than one sub-theme. Sub-themes are therefore not mutually exclusive

### Attitudes towards mental illness

The overall mean score of the eleven questions from the *MICA*-*4* was 28.4/66 (SD = 6.3; Q1 = 24.0, Q2 = 28.0, Q3 = 32.0). These results suggest some gaps in favorable attitudes towards both mental illness and the field of mental health.

Scores based on favorable answers, per individual item, are provided in Table 3. These answers also make apparent gaps in favorable attitudes towards mental health and mental illness. As shown, most PCPs had unfavorable attitudes about: the dangerousness of people with mental health problems, disclosure about mental health problems to colleagues or friends, the PCP’s role in assessing mental health problems in primary care, interactions with people presenting with mental health problems in PCPs’ clinical practice, and personal recovery from a mental health problem. However, PCPs favorably answered concepts relating to the importance of physical health in mental health care, the respectability of being a mental healthcare professional, and respect for people with mental health problems.

**Table 3:** Attitudes towards mental illness and the field of mental health (n = 112)

*MICA-4* items	Favorable answers n (%)
13. If a person with a mental illness complained of physical symptoms (such as chest pain), I would attribute it to their mental illness (R)	108 (96.4)
15. I would use the terms “crazy,” “nutter,” “mad,” etc. to describe to colleagues people with a mental illness who I have seen in my work	101 (90.2)
16. If a colleague told me they had a mental illness, I would still want to work with them	95 (85.6)
1. I just learn about mental health when I have to, and I would not bother reading additional material on it (R)	95 (85.6)
2. People with severe mental illness can never recover enough to have a good quality of life (R)	67 (59.8)
4. If I had a mental illness, I would never admit this to any of my friends because I would fear being treated differently (R)	58 (51.8)
14. General practitioners should not be expected to complete a thorough assessment for people with psychiatric symptoms because they can be referred to a psychiatrist (R)	57 (50.9)
10. I feel comfortable talking to a person with mental illness as I do talking to a person with physical illness	47 (42.0)
7. If I had a mental illness, I would never admit this to my colleagues for fear of being treated differently (R)	46 (41.8)
5. People with mental illness are dangerous more often than not (R)	31 (27.7)
12. The public does not need to be protected from people with mental illness	22 (20.0)

Eleven questions from the original *MICA*-*4* are reported

For reversed scored items (R), suggested answers tend toward the negative (i.e., ‘strongly disagree’ and ‘disagree’), and these negative categories were collapsed into the single category of ‘favorable answers.’ Contrarily, for items not reversed, suggested answers tend toward the positive (i.e., ‘strongly agree’ and ‘agree’), and these positive categories were collapsed into the single category of ‘favorable answers’

Missing data < 5%

### Self-efficacy

PCPs obtained an average overall mean score of 5.1/10 (SD = 1.5; Q1 = 4.0, Q2 = 5.2, Q3 = 6.3) on the self-efficacy questionnaire. PCPs scored higher on scale 1, which regroups concepts related to self-efficacy about detection of mental health problems in primary care (5.8/10, SD = 1.6; Q1 = 4.6, Q2 = 6.0, Q3 = 7.1), than scale 2, which regroups concepts related to self-efficacy about treatment and management of mental health problems in primary care (4.8/10, SD = 1.8; Q1 = 3.6, Q2 = 5.0, Q3 = 6.1). These results suggest gaps in self-efficacy.

Average scores for detection themes on the self-efficacy scale were as follows: 6.0/10 (SD = 1.9; Q1 = 4.7, Q2 = 6.3, Q3 = 7.5) for detection of mental health problems and 5.4/10 (SD = 1.9; Q1 = 3.8, Q2 = 5.6, Q3 = 6.9) for using techniques related to detecting mental health problems. Average scores for treatment and management themes on the self-efficacy scale were as follows: 3.8/10 (SD = 1.8; Q1 = 2.5, Q2 = 3.5, Q3 = 5.0) for treatment by pharmacology, 4.7/10 (SD = 2.1; Q1 = 2.9, Q2 = 4.6, Q3 = 6.1) for treatment by support, 4.7/10 (SD = 2.2; Q1 = 3.0, Q2 = 5.0, Q3 = 6.5) for treatment by psychoeducation, and 5.6/10 (SD = 2.0; Q1 = 4.4, Q2 = 6.6; Q3 = 6.9) for management by developing clinical plans for patients.

Limited perception of confidence in capabilities to detect, treat, and manage mental health problems in primary care is also apparent when looking at responses to each individual item. As shown in Table 4, few PCPs agreed that they felt confident in their capability to detect substance use disorders and psychosis (including schizophrenia). In addition, PCPs in our sample struggled with confidence in their capability to pose a mental health diagnosis, use tools and techniques to detect a mental health problem, and explain a mental health diagnosis to patients.

**Table 4:** Self-efficacy in detecting, treating, and managing mental illness in primary care (n = 112)

Self-efficacy, detection	Agree n (%)
I feel confident in my capability to detect:	
Problems relating to anxiety	92 (82.9)
Depression	83 (74.8)
Suicide/self-harm	60 (54.0)
Problems relating to alcohol use	58 (52.8)
Problems relating to drug use	51 (45.9)
Psychosis (including schizophrenia)	41 (37.3)
I feel confident in my capability to:	
Collect information to detect a mental health problem	73 (66.4)
Explain the diagnosis to patients	55 (49.1)
Diagnose a mental health problem	43 (38.4)
Use tools and techniques to detect a mental health problem	34 (30.4)

Self-efficacy, treatment, and management	Agree n (%)
I feel confident in my capability to provide pharmacological treatment for patients presenting with:	
Problems relating to anxiety	61 (56.5)
Depression	43 (38.7)
Problems relating to alcohol use	11 (10.0)
Problems relating to drug use	11 (10.1)
Psychosis (including schizophrenia)	11 (10.0)
I feel confident in my capability to provide support (ex: active listening) for patients presenting with:	
Depression	84 (75.7)
Problems relating to anxiety	70 (64.3)
Problems relating to drug use	37 (34.0)
Problems relating to alcohol use	36 (32.1)
Psychosis (including schizophrenia)	21 (18.9)
I feel confident in my capability to provide psychoeducation for patients presenting with:	
Depression	58 (52.2)
Problems relating to anxiety	55 (49.5)
Problems relating to alcohol use	36 (33.0)
Problems relating to drug use	32 (28.8)
Psychosis (including schizophrenia)	17 (15.3)
I feel confident in my capability to treat patients having issues relating to:	
Self-harm	31 (27.9)
Suicide	26 (23.4)
I feel confident in my capability to develop a clinical plan for patients presenting with:	
Problems relating to anxiety	56 (50.4)
Depression	51 (45.9)
Problems relating to alcohol use	28 (25.5)
Problems relating to drug use	28 (25.7)
Psychosis (including schizophrenia)	18 (16.3)
I feel confident in my capability to refer my patient	101 (91.8)
I feel confident in my capability to involve family members/friends in the management plan	83 (74.8)
I feel confident in my capability to involve other professionals in the management plan	66 (60.0)

Missing data < 5%

Consistently, PCPs felt less confident in their capability to treat people presenting with symptoms relating to substance use disorders and psychosis (including schizophrenia) than they did with anxiety and depression symptoms, and very few PCPs felt confident in their capability to provide treatment for suicide and/or self-harm. In addition, PCPs in our sample reported very limited confidence in their capability to manage mental health problems in primary care, specifically by developing a clinical plan for patients needing care. Almost all PCPs in our sample felt very confident in their capability to refer people presenting with mental health problems to more specialized settings.

### Characteristics associated with mental health knowledge, attitudes, and self-efficacy

Working in Suburb 3 seemed to be significantly associated with higher levels of mental health self-efficacy (*B* = .859, *p* = .038, *r*^*2*^ = .043). The number of weekly work hours reported by PCPs (*B* =  −.285, *p* = .014, *r*^2^ = .054) and the average number of hours PCPs reported dedicating to mental health care per week (*B* = − 4.608, *p* = .031, *r*^*2*^ = .046) seemed to be significantly associated with more favourable mental health attitudes. In addition, participating in a mental health training during the previous 12 months seemed to be significantly associated with higher levels of mental health knowledge (*B* = .791, *p* = .041, *r*^*2*^ = .037) and higher levels of mental health self-efficacy (*B* = 1.093, *p* = .011, *r*^*2*^= .057).

Mental health self-efficacy seemed to be positively associated with the weekly percentage of PCP-reported clientele engaged in psychoeducation (*B* = .012, *p* = .002, *r*^*2*^ = .090). Mental health knowledge seemed to be significantly negatively associated with the weekly percentage of clientele PCPs reported referring to specialized services (*B* = − .016, *p* = < .001, *r*^*2*^ = .128).

## Discussion

We report on PCPs’ knowledge and attitudes about mental health, as well as their sense of self-efficacy, prior to the implementation of a mental health training program, and we highlight variables that are associated with these competencies. Results show that PCPs in our sample detect, treat, and manage mental illness in primary care, but limitations to their involvement are apparent.

To the authors’ knowledge, this article is the first to detail mental health knowledge, attitudes, and perceived self-efficacy, as well as characteristics that may be associated with such competencies, among PCPs working in the Greater Tunis area of Tunisia. Such results are timely given the following factors: the current push in global mental health to use non-specialists in mental health care [2–4, 7]; the need to develop and design tailored medical education curricula and continuing medical education programs, severely lacking in LMICs [8, 44, 45, 70–73]; and the scarcity of mental health research in Tunisia, also a reality in other LMICs [74, 75].

Findings in our sample, as compared to others, raise a prominent issue: PCPs show gaps in knowledge about mental illness, hold certain negative beliefs about mental illness and the field of mental health, and lack confidence in specific capabilities [11–13, 15, 17, 23–25, 27, 42]. These limits are important to highlight because they may hinder mental health care encouraged in non-specialized settings [2–4] and thus the full potential of non-specialists’ involvement in the field of mental health [44]. However, worthy of note is that this lack of perceived confidence in specific capabilities may be appropriate, since it does somewhat match and reflect certain levels of knowledge and unfavorable beliefs scored by PCPs in our sample prior to training.

Most incorrect responses reported by PCPs in our sample on the knowledge questionnaire relate to substance use disorders and suicide/self-harm. In addition, PCPs in our sample consistently scored lower on perceived self-efficacy related to detection, treatment, and management of substance use disorders, suicide/self-harm, and psychosis than they did when asked similar questions about depression and problems relating to anxiety. These incorrect answers and lower levels of confidence in capabilities for specific disorders may not be surprising; non-specialists such as PCPs often continue to favour consultations for depression and/or anxiety, despite some apparent knowledge [11–13, 15] and confidence gaps [42], over those they deem more complex disorders [11, 12, 21, 22, 76, 77]. Such notions may also be confirmed in our sample: PCPs estimated that the highest percentage of mental health consultations per week were for symptoms relating to depression and anxiety. However, what we found surprising was that despite PCPs’ low scores on perceived self-efficacy related to psychosis, their sub-theme average for knowledge about this condition was one of the highest. Thus, there appears to be a gap between PCPs’ theoretical knowledge about psychosis and their confidence in skills related to detection, treatment, and management of this disorder in clinical practice. Interestingly, the opposite finding was reported by Cowan and colleagues [13]; while most PCPs in their sample in India reported a high degree of self-perceived competence in detecting symptoms of psychosis, they were unable to accurately name three common symptoms related to this condition. Discrepancies between theoretical knowledge of mental health and perceived confidence in mental health capabilities may be important to highlight; having high perceived confidence in specific capabilities, if there are deficits of knowledge in mental health, can potentially spell poorer clinical care and even danger to patients.

The ongoing drafting of national substance use and suicide prevention strategies, as well as the implementation of national anti-stigma campaigns monitored by the *Committee for Mental Health Promotion* at the level of the Ministry of Health, aim to further promote the recognition of substance use disorders, self-harm, and suicide in Tunisia, as these conditions continue to be heavily stigmatized in the country [23, 39, 78]. Stigmatization may lead to disinterest, especially among primary care staff, underdiagnoses and/or under-reporting, limited options for treatment beyond specialized care, and few research initiatives in the field [79, 80]. Thus, referral of patients presenting with substance use disorders, suicide/self-harm, and psychosis (including schizophrenia) is still very common in Tunisia [78], limiting PCPs’ contact and involvement with these conditions in primary care, as shown in our sample.

We found several characteristics among our sample that seemed to be associated with PCPs’ competencies. In several cases, such competencies seemed to be associated with levels of clinical practice. Findings from simple linear regression models thus seem to reinforce two important aspects in mental health capacity-building. The first aspect seems to be the importance of providing healthcare professionals the opportunity for positive social contact, interaction, and involvement with people living with mental health issues. Research has shown that this type of contact, interaction, and involvement is effective in decreasing negative beliefs about mental illness [81, 82], building confidence with such clientele, and consequently decreasing healthcare professionals’ reluctance to engage in mental health care in clinical practice [26, 50, 83]. Therefore, in parallel to anti-stigma campaigns and the institutionalization of best mental health practices through the drafting of national substance use and suicide prevention strategies in Tunisia, ways to encourage PCPs’ positive social interactions and involvement with people presenting with mental health issues in primary care settings, even those they deem to be more complex, would likely be beneficial. Such initiatives may include continuing mental health education programs with access to practica, and, for support with challenging cases, ongoing supervision. Second, these training programs may be tailored to specific governorates given that our findings seem to suggest that work location may be associated with levels of PCPs’ mental health self-efficacy. Tailoring training programs and curricula, as well as integrating interactive and practical components to such programs were also suggested by authors who identified gaps in PCPs’ mental health knowledge, attitudes, and self-efficacy in other LMICs [7, 12, 15, 24, 84].

Finally, by using the *MICA*-*4*, we were able to identify negative attitudes towards mental illness and the field of mental health among our sample, which are also common among other non-specialists working in LMICs [14, 19, 23–25, 27–31, 33–36]. While the *MICA*-*4* has been used in other contexts [35, 36, 85–90], internal consistency and some item-total correlations generated using the sixteen-item scale were poor in our sample. These poor results lead us to question its suitability to assess PCPs’ attitudes towards mental illness and the field of mental health in the Greater Tunis area of Tunisia and in French-speaking LMICs more generally. However, we were able to explore mental health stigma using eleven questions of the *MICA*-*4* with a Cronbach’s alpha of .608, an increase from our initial assessment with the original sixteen items. We were thus able to show that most PCPs in our sample held exaggerated negative beliefs about the dangerousness of people with mental health problems. More specifically, most PCPs in our sample did not answer the following questions favorably: (1) people with mental illness are dangerous more often than not; and (2) the public does not need to be protected from people with mental illness. This fear, an effect of stigmatization common in other low-resource settings [12, 27–30], may help explain, in part, why most PCPs in our sample (91.8%) reported feeling very confident in their capability to refer patients to more specialized care, which, in Tunisia, is frequently remote from the homes and communities of patients [23, 58]. Confidence in referral to specialized care also seems to be concretely translated into self-reported practice; per week, PCPs refer most people consulting for mental health issues to specialized resources (55.6; SD = 30.8; Q1 = 30.0, Q2 = 50.0, Q3 = 80.0).

While it is encouraging to note that PCPs in our sample do engage in mental health care, identified gaps in mental health knowledge, attitudes, and self-efficacy, as well as associations between certain characteristics and such competencies uncovered by simple linear regression models, seem to support two mental health initiatives confirmed in Tunisia: the implementation of a mental health training program in the Greater Tunis area, under the auspices of the *Committee for Mental Health Promotion* [23, 39, 78], and the recent inclusion of a mandatory (previously optional) 2-month internship in post-graduate medical curricula to train future PCPs in effective mental health detection, treatment, and management [91]. Continuing mental health training and a mandatory mental health internship with access to support and guidance to encourage positive contact and interaction with people living with mental health issues are thus strategies that Tunisia has adopted to help build non-specialists’ competencies in mental health. These also align with internationally supported ways to help target the mental health treatment gap and further integrate mental health into primary and community-based settings [2–4].

### Strengths and limitations

There were methodological strengths and limitations to the study. First, the goal of the trial, in which this paper is inscribed, was not to generalize results to all PCPs working in Tunisia, but to see if the training program worked before considering larger-scale implementation. Hence, we cannot ascertain if our results are generalizable to all PCPs in Tunisia. However, we assume that these competencies and gaps may be similar to those of public sector PCPs working in other areas of Tunisia who would agree to participate in a mental health training. Second, results are based on self-reports, not on observed behaviour or review of patient records. Therefore, we cannot determine whether responses are driven by social desirability. However, the honesty reported by PCPs on questions related to the dangerousness of people with mental health problems and to the public’s need for protection from people with mental illness seems to indicate authenticity. In addition, these questions show very little missing data (< 2%). Third, given the nature of self-report questionnaires, practice characteristics reported by PCPs in our sample should be considered an approximation. Fourth, scales used to assess knowledge and self-efficacy were not previously validated. However, we believe a strength of this paper is the provision of some measures of reliability for these scales, based on our sample from the Greater Tunis area, which proved to be acceptable. Fifth, reliability measures for the *MICA*-*4* based on our sample complement the literature on the *MICA*-*4*’s psychometric properties, a strength of this paper given that the scale’s authors suggest considering its applicability across other samples [62]. However, it is important to note that while the *MICA*-*4* had acceptable internal consistency in a previous study [62], it did not show results that were as promising in our sample. We therefore aimed to improve internal consistency by reporting solely on eleven items from the original scale, which limited our ability to compare the overall score with other studies using all sixteen questions. Further research is needed to assess whether possible sub-scales are identifiable in our sample and comparable to the ones identified by the authors of the scale [62]. Finally, we believe that further research is needed to explore the associations among socio-demographic and practice characteristics, as well as PCPs’ competencies.

## Conclusion

Involving non-specialists such as PCPs in the care of people living with mental health problems is encouraged internationally as one of the initiatives to address the mental health treatment gap in LMICs. While non-specialists do engage in mental health care, it is not uncommon for them to lack specific mental health competencies used to detect, treat, and manage mental health issues in non-specialized settings. This paper reported on mental health knowledge, attitudes, and self-efficacy among a sample of PCPs working in the Greater Tunis area, prior to the implementation of a mental health training program. It also highlighted associations between socio-demographic/practice characteristics and such competencies. Findings may encourage other LMICs to assess the current mental health competencies of non-specialists, information that may be used to develop specific and tailored mental health initiatives to further promote their involvement in effective mental health care, as well as the integration of mental health into primary and community-based settings.

## Additional file

**Additional file 1.** Item-total correlation and Cronbach's alpha for the *MICA-4*.

## Abbreviations

MNS: mental, neurological, and substance use
LMICs: low- and middle-income countries
PCPs: primary care physicians
WHO: World Health Organization
mhGAP: Mental Health Gap Action Programme
IG: intervention guide
ICC: Intraclass Correlation Coefficient
IC: confidence intervals
MICA: Mental Illness Clinicians’ Attitudes
SD: standard deviation
