# Supplementary material for: Mental health knowledge, attitudes, and self-efficacy among primary care physicians working in the Greater Tunis area of Tunisia
Source: Int J Ment Health Syst. 2018 Oct 26;12:63. doi: 10.1186/s13033-018-0243-x (PMC6203218; doi:10.1186/s13033-018-0243-x)
Supplement: Supplementary file 1 — Additional file 1. Item-total correlation and Cronbach's alpha for the MICA-4. [file 13033_2018_243_MOESM1_ESM.docx]

|  | Original scale | | Test 1 | | Test 2 | | Test 3 | | Test 4 | | Test 5 | |
| --- | --- | --- | --- | --- | --- | --- | --- | --- | --- | --- | --- | --- |
| *MICA-4 items* | Item-total correlation coefficient | Cronbach’s alpha if item excluded | Item-total correlation coefficient | Cronbach’s alpha if item excluded | Item-total correlation coefficient | Cronbach’s alpha if item excluded | Item-total correlation coefficient | Cronbach’s alpha if item excluded | Item-total correlation coefficient | Cronbach’s alpha if item excluded | Item-total correlation coefficient | Cronbach’s alpha if item excluded |
| 1. I just learn about  mental health when I  have to, and I would  not bother reading  additional material on  it. (R).  2. People with severe  mental illness can  never recover enough  to have a good  quality of life. (R)  3. Working in the  mental health field  is just as respectable  as other fields of  health and social  care.  4. If I had a mental  illness, I would  never admit this to  any of my friends  because I would fear  being treated  differently. (R)  5. People with mental  illness are dangerous  more often than not.  (R)  6. Health/social care  staff know more  about the lives of  people treated for a  mental illness than do  family members  and friends. (R)  7. If I had a mental  illness, I would  never admit this to  my colleagues for  fear of being treated  differently. (R)  8. Being a  health/social care  professional in the  area of mental  health is not like  being a real  health/social care  professional. (R)  9. If a senior  colleague instructed  me to treat people  with mental illness in  a disrespectful  manner, I would not  follow their  instructions.  10. I feel comfortable  talking to a person  with mental illness as  I do talking to a  person with physical  illness.  11. It is important  that any health/social  care professional  supporting a person  with mental illness  also ensures that their  physical health is  assessed.  12. The public does  not need to be  protected from people  with mental  illness.  13. If a person with a  mental illness  complained of  physical symptoms  (such as chest pain), I  would attribute  it to their mental  illness. (R)  14. General  practitioners should  not be expected to  complete a thorough  assessment for people  with psychiatric  symptoms because  they can be  referred to a  psychiatrist. (R)  15. I would use the  terms “crazy,”  “nutter,” “mad,” etc.  to describe to  colleagues people  with mental illness  that I have seen in  their work. (R)  16. If a colleague told  me they had a  mental illness, I  would still want to  work with them. | 0.357  0.146  0.030  0.314  0.389  -0.019  0.222  0.066  0.120  0.217  0.016  0.156  0.255  0.252  0.259  0.277 | 0.478  0.511  0.528  0.471  0.455  0.552  0.495  0.534  0.518  0.496  0.529  0.511  0.504  0.487  0.493  0.492 | 0.468  0.166  0.029  0.292  0.377  -  0.200  0.033  0.168  0.235  0.010  0.158  0.275  0.288  0.303  0.289 | 0.511  0.542  0.561  0.514  0.495  -  0.536  0.577  0.543  0.528  0.563  0.547  0.535  0.515  0.521  0.525 | 0.364  0.186  0.025  0.296  0.286  -  0.212  0.031  0.146  0.245  -  0.141  0.278  0.294  0.301  0.308 | 0.523  0.550  0.573  0.525  0.505  -  0.545  0.590  0.559  0.538  -  0.563  0.546  0.526  0.533  0.534 | 0.380  0.207  -  0.315  0.382  -  0.215  0.031  0.141  0.245  -  0.125  0.257    0.303  0.295  0.308 | 0.532  0.558  -  0.533  0.518  -  0.557  0.603^a^  0.573  0.550  -  0.579  0.559  0.536  0.545  0.545 | 0.342  0.249  -  0.384  0.314  -  0.253  -  0.116  0.239  -  0.121  0.281  0.304  0.293  0.330 | 0.566  0.578  -  0.546  0.563  -  0.578  -  0.608  0.582  -  0.611  0.584  0.566  0.573  0.569 | 0.360  0.243  -  0.424  0.306  -  0.275  -  -  0.217  -  0.109  0.247  0.309  0.280  0.357 | 0.572  0.590  -  0.544  0.576  -  0.584  -  -  0.600  -  0.627  0.596  0.575  0.585  0.574 |
| *Cronbach’s alpha*  *for the scale* |  | **0.521** |  | **0.552** |  | **0.563** |  | **0.573** |  | **0.598^a^** |  | **0.608** |

R = reversed score item

^a^ = This incongruous result is due to how SPSS 25 computes the Cronbach's alpha if deleted.
